# Safety and effectiveness of intra-articular injection of a highly cross-linked hyaluronic acid, LBSA0103 (Synovian): Results from a post-marketing surveillance study in South Korea

**DOI:** 10.1371/journal.pone.0287222

**Published:** 2023-06-22

**Authors:** Jae Gyoon Kim, Kang-Il Kim, Ki-Bong Park, Yong-Geun Park, Ji Hoon Bae, Young-Jin Seo, Jong-Keun Seon, Oog Jin Shon, Ji Hyun Ahn, Lih Wang, Joon-Ho Wang, Eui Sung Choi, Jeong-Ku Ha, Hyuk-Soo Han, Sang Won Moon

**Affiliations:** 1 Department of Orthopedic Surgery, Korea University College of Medicine Ansan Hospital, Gyeongki-do, Republic of Korea; 2 Department of Orthopedic Surgery, School of Medicine, Kyung Hee University, Seoul, Republic of Korea; 3 Department of Orthopedic Surgery, Good Samsun Hospital, Busan, Republic of Korea; 4 Department of Orthopedic Surgery, Jeju National University Hospital, Jeju National University College of Medicine, Jeju, Republic of Korea; 5 Department of Orthopedic Surgery, Korea University College of Medicine, Korea University Guro Hospital, Seoul, Republic of Korea; 6 Department of Orthopedic Surgery, Hallym University Dongtan Sacred Heart Hospital, Hwaseong, Republic of Korea; 7 Department of Orthopedic Surgery, Chonnam National University Hwasun Hospital and Medical School, Jeonnam, Republic of Korea; 8 Department of Orthopedic Surgery, School of Medicine, Yeungnam University Hospital, Daegu, Republic of Korea; 9 Department of Orthopedic Surgery, Kangbuk Samsung Hospital, School of Medicine, Sungkyunkwan University, Seoul, Republic of Korea; 10 Department of Orthopedic Surgery, Dong-A University College of Medicine, Dong-A University Hospital, Busan, Korea; 11 Department of Orthopedic Surgery, Sungkyunkwan University School of Medicine, Seoul, Korea; 12 Department of Orthopedic Surgery, Chungbuk National University College of Medicine, Cheongju, Korea; 13 Department of Orthopedic Surgery, Inje University Seoul Paik Hospital, Inje University College of Medicine, Seoul, Republic of Korea; 14 Department of Orthopedic Surgery, Seoul National University College of Medicine, Seoul, Republic of Korea; 15 Department of Orthopedic Surgery, Inje University Haeundae Paik Hospital, Busan, Korea; UNITED STATES

## Abstract

This study aimed to assess the safety and effectiveness of the highly cross-linked hyaluronic acid—LBSA0103—in patients with knee osteoarthritis (OA) as per the prescribing information (PI) in South Korea. A total of 3,140 subjects aged ≥19 years were enrolled in this post-marketing surveillance (PMS) study from 2013 to 2019. The subjects received one or two injections of LBSA0103. The median duration of follow-up was 308 days. Adverse events (AEs), adverse drug reactions (ADRs), and serious AEs (SAEs) were monitored. Effectiveness was evaluated based on an index of effectiveness in accordance with the guidelines established by the Ministry of Food and Drug Safety and using a 100-mm visual analog scale (VAS) for weight-bearing pain. Overall, 250 subjects (7.96%) experienced 292 AEs and of these, unexpected AEs occurred in 114 subjects (3.63% [95% CI: 3.00–4.35]). Injection site pain was the most frequent AE reported by 81 subjects (2.58% [95% confidence intervals (CI): 2.05–3.20]). One hundred subjects experienced 108 ADRs (3.18% [95% CI: 2.60, 3.86]) and 15 unexpected ADRs were experienced by 13 subjects (0.41% [95% CI: 0.22–0.71]). Seventeen subjects experienced 22 SAEs (0.54% [95% CI: 0.32–0.87]) during the entire PMS period, and all were considered “unlikely” related to the study drug. Most AEs were mild in terms of severity and resolved during the study period. LBSA0103 was also effective in relieving symptomatic pain in knee OA patients. The condition in more than 80% of the subjects was considered to be improved when assessed by the investigators. LBSA0103 resulted in a significant reduction in the mean VAS score at 12 weeks after the first and second injections (24.79 (± 20.55) mm and 17.63 (±12.31) mm, respectively; *p*<0.0001). In conclusion, LBSA0103, used for the treatment of knee OA in a real-world setting, was well tolerated, with an acceptable safety profile and consistent therapeutic effect.

## 1. Introduction

Knee osteoarthritis (OA) is a progressive joint disease characterized by cartilage degeneration and inflammation [1]. Knee OA is one of the leading causes of pain and functional disability in the elderly population [2]. There are different treatment options available for knee OA [1, 2]; however, none are effective in preventing disease progression. To date, pharmacological approaches have been directed toward symptomatic pain relief and improvement in physical function [3, 4]. Among the pharmacologic therapies available, intra-articular (IA) hyaluronic acid (HA) injections, also known as viscosupplementation, have been widely used to treat knee OA [3–7].

HA is a molecule that is intrinsically found within the knee joint, where it provides viscoelastic properties to the synovial fluid [3]. The notion of supplementing HA in knee OA is based on the fact that the concentration and molecular weight (MW) of HA are reduced in these patients, resulting in degradation of the mechanical and viscoelastic properties of the endogenous synovial fluid [8, 9]. The low concentration and MW of HA have also been strongly correlated with pain [9]. The exact mechanism of action by which IA-HA alleviate pain in OA joints is not yet fully understood. In*-*vitro and in*-*vivo studies suggest that exogenous HA stimulates the endogenous production of additional hyaluronate, reduces inflammatory mediator levels in the synovial fluid, reduces chondrocyte apoptosis, increases chondrocyte proliferation, and enhances proteoglycan and glycosaminoglycan synthesis [6]. There is also evidence suggesting that HA with a higher MW (HMW) provides greater anti-inflammatory and proteoglycan synthesis effects and ensures joint lubrication and viscoelasticity maintenance, thereby enhancing the therapeutic effect [6]. Cross-linking is also known to provide longer-lasting efficacy to HAs by increasing the viscoelasticity and resistance to degradation in the joint space [6]. However, there is evidence that suggests that cross-linked HAs is associated with an increased incidence of severe acute inflammatory reaction, which is clinically different from the reactions observed in non-cross-linked HAs. Rare cases of pseudosepsis have been reported in subjects receiving cross-linked HMW-HAs [10–16]. Some studies have attributed this increased immunogenicity to the source of HAs [10, 15], whereas other studies have speculated that the cross-linking structure is the cause of pseudoseptic reactions [11, 16].

The current recommendations provided by clinical practice guidelines for intra-articular (IA) hyaluronic acid (HA) treatment for knee OA are highly inconsistent [5, 7, 17]. Despite the ambiguity in the recommendation concerning IA-HA as a treatment modality, IA-HA injections, have been widely used to treat symptomatic knee OA. The tolerable safety profile and long-lasting effects make it an attractive treatment option for patients with knee OA. Furthermore, Osteoarthritis Research Society International (ORASI) recently changed their stance in 2019, giving a “conditional recommendation for the use of IA-HA for a long-term effect where multiple intra-articular corticosteroids are contraindicated” [18] from “uncertain” for the recommendation for the use of IA-HA in their 2014 guideline [4]. The guideline claimed that IA-HA was associated with symptom improvement beyond 12 weeks and demonstrated a favorable safety profile [18].

LBSA0103 (Synovian^TM^; LG Chem, Ltd., Iksan, Republic of Korea) is an HMW-HA (≥10 million dalton) cross-linked with 1,4-butanediol diglycidyl ether (BDDE). It is synthesized by bacterial fermentation using *Streptococcus zooepidemicus*. The cross-linking agent, BDDE, is known to have a significantly lower toxicity than other ether bond cross-linking agents (e.g., divinyl sulfone [DVS]) and is biodegradable [19, 20]. BDDE has reactive epoxide groups on either side of the chain, but these groups form stable ether bonds with alcohol in the HA backbone and are neutralized, making the amount of unreacted BDDE negligible [20]. The source from which LBSA0103 is derived and the cross-linking agent utilized make LBSA0103 a safe alternative to other modified HMW-HA preparations. Likewise, the safety and effectiveness of LBSA0103 have been well established from studies conducted during the product’s clinical development [21, 22]. This PMS study aimed to assess the safety and effectiveness of LBSA0103 when administered to Korean patients with knee OA in accordance with the Korean prescribing information (PI), in a real-world clinical setting.

## 2. Materials and methods

### 2.1 Study design

**Fig 1:**
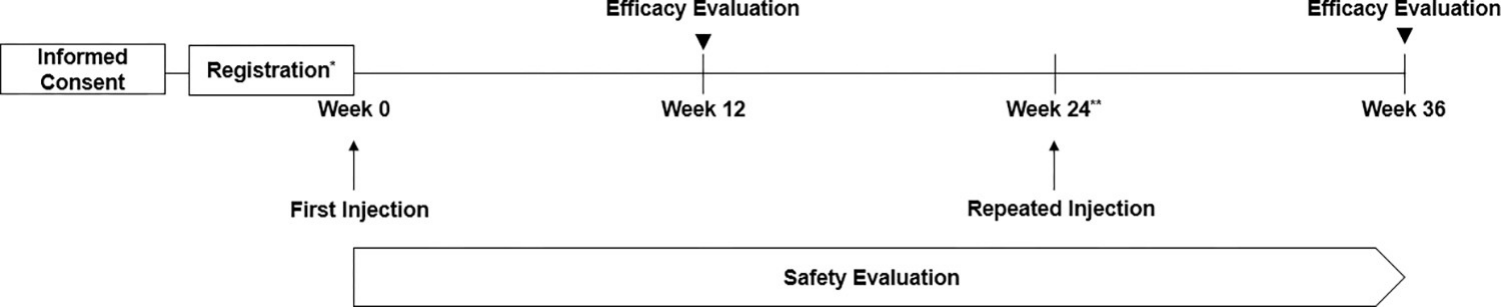
Flow of the study. * If the criteria for inclusion/exclusion of subjects are met and all necessary information has been collected before the administration, the registration date (the date written consent and permission to use data are obtained) and the administration date may be the same. ** Second injection was at least 6 months after the first injection.

This was a prospective, non-interventional, multicenter, PMS study conducted from October 15, 2013, to October 14, 2019, to assess the safety and efficacy of LBSA0103 for the treatment of knee OA. The study was conducted in accordance with the requirements of the Pharmaceutical Affairs Law and the ministerial ordinance of *Re-examination Standards for New Drugs*, *Etc*. enforced by the Ministry of Food and Drug Safety (MFDS), which mandates the PMS of new drugs or new molecular entities. LBSA0103 was subjected to a mandatory PMS on a minimum of 3,000 subjects aged ≥19 years over a study period of 6 years. Investigators of the contracted institutions registered subjects who were prescribed LBSA0103 and provided data on the safety and effectiveness of the study product for each treated patient. Data on subject demographics, such as age, sex, height, weight, preexisting/concurrent medical conditions (including hepatic and renal impairments), presence of an allergy, and preexisting/concomitant medication use and therapy (any medications used or therapies received 24 weeks prior to the IA injections of LBSA0103 and during the follow-up period), were collected at registration. Each patient received either one or two IA injections of LBSA0103 and was followed up to 12 weeks after each injection. Second injections were only administered to those who required them at 24 weeks after the first injection, in accordance with the approved PI. Data were gathered as part of routine clinical monitoring and collected in a standardized case report form upon registration and at every visit thereafter (Fig 1).

### 2.2 Study population

Patients who signed the consent form and met all the inclusion and exclusion criteria were enrolled in the study. Participants aged ≥19 years, diagnosed with knee OA, and in whom treatment with LBSA0103 was decided according to the locally approved PI were included in the study. The major exclusion criteria were previous administration of LBSA0103, history of allergic reactions to any component of the study drug, and infection or severe inflammation in the synovial joint where the drug was to be administered or at the injection site. Subjects aged ≥65 years as well as those with renal or hepatic impairment were classified as a special population, and subgroup analyses were conducted to further evaluate the safety and effectiveness in this population.

### 2.3 Ethics statement

This PMS study was conducted in accordance with the regulatory requirements and regulations of the MFDS. Ethical approval by an institutional review board was not required for this study; however, ethical approval was obtained by study centers with their own institutional rules. The subjects provided written informed consent for the collection and handling of personal data and safety information before study enrollment.

### 2.4 Safety assessments

To evaluate the safety of LBSA0103, AEs and abnormal laboratory findings, either voluntarily reported by subjects or identified by the treating physician during follow-ups, were collected and assessed. There were no prespecified AEs of special interest; all AEs that occurred during the observation period were included and assessed in accordance with the guideline established by the MFDS. An ADR was defined as “adverse, unintended reactions from normal administration/use of the pharmaceutical, for which the causal relationship with the particular pharmaceutical cannot be excluded,” and an unexpected AE was defined as “an AE whose nature, severity, specificity, or outcome is not consistent with the term or description used in the PI in Korea.” The causal relation of AEs was categorized as certain, probable, possible, unlikely, conditional/unclassified, or unassessable/unclassifiable, as assessed by the investigator. The intensity of AEs was recorded as mild, moderate, or severe. Serious AEs (SAEs) were referred as “any untoward medical occurrence that at any dose results in death or life-threatening condition, hospitalization, permanent disability, congenital anomaly/birth defect or a medically important event or reaction.”

### 2.5 Effectiveness assessments

Effectiveness was based on subjective measures in accordance with the guidelines established by the MFDS. Effectiveness was evaluated by investigators as one of four outcomes: improved (improvement in symptoms), unchanged (no change compared with that noted at pre-administration), worsened (worsening of symptoms compared with that noted at pre-administration), and undeterminable (in situations where assessment is not possible). Since LBSA0103 was approved for the symptomatic relief of knee OA, “improved” was assessed as “effective.” Subjective effectiveness was measured 12 weeks after the last injection. Effectiveness was also evaluated based on changes in WBP using a standard 100-mm visual analog scale (VAS) [21–23]. The WBP using the VAS was measured at every visit, and the effectiveness outcome was evaluated in terms of changes in WBP at 12 weeks after the last injection.

### 2.6 Statistical analysis

The sample size was predefined to ensure that there would be at least 3,000 safety evaluable subjects for the entire study duration, in accordance with the *Re-examination Standards for New Drugs*, *Etc*. Safety analysis was based on the evaluable population, defined as subjects who received LBSA0103 and were followed up for safety assessment at least once. AEs and SAEs, based on the World Health Organization-Adverse Reactions Terminology dictionary [24], were analyzed and are presented as 95% confidence intervals (CIs). Effectiveness analyses were based on the evaluable population, defined as subjects who received LBSA0103 and completed the effectiveness assessment. The frequency and percentage of subjects with effectiveness are presented. The mean change in 100-mm VAS scores at 12 weeks after the last administration was presented and tested using the paired *t*-test or Wilcoxon’s signed rank test.

Subgroup analyses (defined by age, preexisting/concurrent medical condition, preexisting/concomitant medication, etc.) were conducted using Pearson’s chi-squared test or Fisher’s exact test to determine the significant factors associated with the effectiveness and incidence rate of AEs. These data were further analyzed using logistic regression models to identify factors that were significantly correlated with the safety and effectiveness of the study drug. All variables that were significant in the simple logistic regression test were included in the multiple logistic regression model. Factors associated with the incidence of AEs or the effectiveness of the study drug are presented as odds ratios (ORs) and 95% CIs. All statistical analyses were performed using the SAS version 9.4 software program (SAS Institute, Inc., Cary, NC, USA).

## 3. Results

### 3.1 Patient disposition

**Fig 2:**
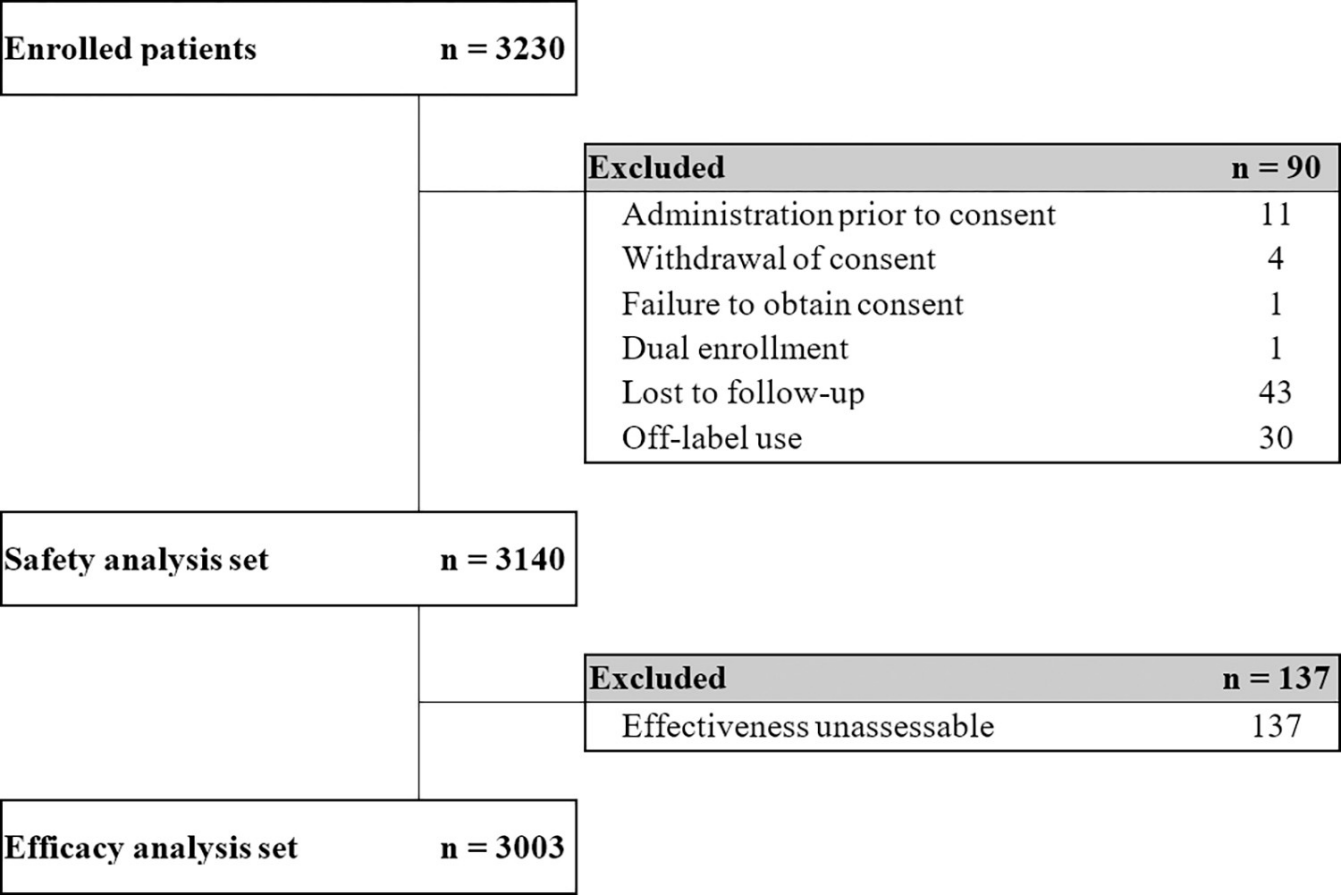
Flow of subjects throughout the study.

A total of 3,230 subjects were enrolled across 145 institutions during the 6-year PMS period. Of these, 90 subjects were excluded, and 3,140 were included in the safety analysis. For analysis of effectiveness, 137 subjects not considered assessable were excluded, and data from the remaining 3,003 subjects were analyzed. The main reasons for exclusion in the effectiveness analysis set were loss to follow-up and effectiveness data not assessable based on the investigator’s judgment. Patient disposition is shown in Fig 2.

### 3.2 Patient demographics and clinical characteristics

The patient demographics and clinical characteristics at baseline are summarized in Table 1. The mean (± standard deviation [SD]) age of subjects at baseline was 64.03 (±10.51) years, and the body mass index (BMI) was 24.75 ± 3.31 kg/m^2^. There were more female participants (73.60%) than males. A similar proportion of subjects with knee OA were affected unilaterally (right, 29.59%; left, 29.01%), and 41.40% of the subjects were affected bilaterally. The mean duration of the disease was 17.57 (± 35.37) months. A total of 107 subjects (3.41%) received a second injection of LBSA0103, and of these, one subject received a third injection, all of them at intervals of at least 24 weeks. Among the 3,140 subjects, 8.98% and 41.05% had preexisting and concurrent medical conditions, respectively. In addition, ≤1.5% of subjects had renal or hepatic impairment. The median duration of follow-up was 308 days.

**Table 1:** Baseline characteristics.

		N = 3,140	
		n	(%)
**Sex**			
	Male	829	(26.40)
	Female	2,311	(73.60)
**Age (years)** ^*^			
	Mean ± SD	64.03 ± 10.51	
	Median	64.00	
**Geriatrics** *			
	≥65	1,609	(51.26)
	<65	1,530	(48.74)
**BMI (kg/m** ^**2**^ **)** ^†^			
	Mean ± SD	24.75±3.31	
	Median	24.52	
**Affected knee OA**			
	Right	929	(29.59)
	Left	911	(29.01)
	Both	1,300	(41.40)
**Duration of knee OA (months)**			
	Mean ± SD	17.57 ± 35.37	
	Median	2.17	
**No. of injection**			
	Once	3,033	(96.59)
	Twice^‡^	107	(3.41)
**Presence of allergy**			
	Yes	77	(2.45)
	No	2,752	(87.64)
	Unknown	311	(9.90)
**Preexisting medical condition**			
	Yes	282	(8.98)
	No	2,858	(91.02)
**Concurrent medical condition**			
	Yes	1,289	(41.05)
	No	1,851	(58.95)
**Renal impairment**			
	Yes	39	(1.24)
	No	3,101	(98.76)
**Hepatic impairment**			
	Yes	49	(1.56)
	No	3,091	(98.44)
**Other medical condition (preexisting/concurrent)** ^**£**^			
	Yes	1,375	(43.79)
	No	1,765	(56.21)
**Preexisting/concomitant medication**			
	Yes	2,078	(66.18)
	No	1,062	(33.82)
**Prior/concomitant therapy**			
	Yes	171	(5.45)
	No	2,969	(94.55)

BMI, body mass index; SD, standard deviation

* Data acquired from 3,139 subjects were used in the analysis.

^†^ Data acquired from 1,178 subjects were used in the analysis.

^‡^ Of these, one subject received a third injection.

^£^Medical conditions other than hepatic or renal impairment.

#### 3.2.1 Safety analysis

Overall, 250 subjects (7.96%) experienced 292 AEs, and 100 subjects experienced 108 ADRs, with an incidence rate of 3.18%; of these, 15 unexpected ADRs were experienced by 13 subjects (0.41%) (Table 2). Injection site pain was the most common ADR (2.20%) and the injection site reaction was the most frequently reported unexpected ADR (0.19%) (Tables 3 and 4**)**. No subject experienced serious ADRs during the entire study period. Among the subjects who experienced SAEs, three (0.10%) died due to pneumonia, ovarian carcinoma, and cardiac arrest, but none of the deaths were related to the study drug. Pneumonia is an infection of the lungs, which cannot be conclusively attributed to the study drug. The subject who died of ovarian carcinoma showed symptoms related to this disease before the administration of the study drug. The exact cause of death was unknown for the subject who died of cardiac arrest; however, the causal relationship was assessed as “unlikely” related to the study drug.

**Table 2:** Summary of adverse events: Safety set.

	N = 3,140			
	No. of subjects (%)		95% CI^‡^	No. of events
Total AE	250	(7.96)	[7.04, 8.96]	[292]
AE–injection site reaction	117	(3.73)	[3.09, 4.45]	[121]
AE–other than injection site reaction	143	(4.55)	[3.85, 5.34]	[171]
ADR	100	(3.18)	[2.60, 3.86]	[108]
SAE	17	(0.54)	[0.32, 0.87]	[22]
Serious ADR	0	(0.00)	[0.00, 0.12]	[0]
Unexpected AE	114	(3.63)	[3.00, 4.35]	[133]
Unexpected ADR	13	(0.41)	[0.22, 0.71]	[15]

AE, adverse event; ADR, adverse drug reaction; SAE, serious adverse event

^‡^ 95% CI calculated using the Clopper-Pearson method

**Table 3:** Summary of adverse drug reactions: Safety set.

Preferred term (or included term)		N = 3,140		
		Number of subjects (%)		Number of events
	Injection site pain	69	(2.20)	[69]
	Injection site burning^£^	13	(0.41)	[13]
	Injection site reaction	6	(0.19)	[6]
	Injection site swelling^£^	4	(0.13)	[4]
	Paresthesia	2	(0.06)	[2]
	Application site edema	1	(0.03)	[1]
	Joint stiffness^£^	1	(0.03)	[1]
	Injection site rash	1	(0.03)	[1]
	Dyspepsia	1	(0.03)	[1]
	Fullness abdominal ^£^	1	(0.03)	[1]
	Arthralgia	1	(0.03)	[1]
	Leg pain	1	(0.03)	[1]
	Oedema legs ^£^	1	(0.03)	[1]
	Pain in limbs^£^	1	(0.03)	[1]
	Heaviness in limbs^£^	1	(0.03)	[1]
	Dyspnea	1	(0.03)	[1]
	Pruritus	1	(0.03)	[1]
	Hordeolum^£^	1	(0.03)	[1]
	Micturition frequency	1	(0.03)	[1]

Dictionary: The World Health Organization-Adverse Reactions Terminology version 092

^£^Included term

**Table 4:** Summary of unexpected adverse drug reactions: Safety set.

Preferred term (or included term)		N = 3,140		
		Number of subjects (%)		Number of events
	Injection site reaction	6	(0.19)	[6]
	Fullness abdominal^£^	1	(0.03)	[1]
	Joint stiffness^£^	1	(0.03)	[1]
	Injection site rash	1	(0.03)	[1]
	Oedema legs ^£^	1	(0.03)	[1]
	Heaviness in limbs^£^	1	(0.03)	[1]
	Pruritus	1	(0.03)	[1]
	Dyspnea	1	(0.03)	[1]

Dictionary: The World Health Organization-Adverse Reactions Terminology version 092

^£^ Included term

#### 3.2.2 Subgroup analysis

The safety findings in special populations were generally similar to those in the overall population. However, the incidence rate of AEs was significantly higher in subjects aged ≥65 years than in those aged <65 years (9.28% vs. 6.71%, *p* = 0.0079). The incidence rate of ADRs in subjects aged ≥65 years (3.33%) was similar to that in the overall population (3.18%). No significant differences were noted in the incidence rate of AEs between subjects with normal renal or hepatic function and those with renal or hepatic impairment (*p* = 0.1236 and *p* = 0.5893 for renal and hepatic impairments, respectively).

Moreover, the safety profile of 107 subjects (3.41%) who received a second injection of LBSA0103 was evaluated, and 15 subjects (14.02%) experienced AEs; the subjects who received two injections of LBSA0103 were associated with a significantly higher incidence rate of AEs compared with subjects who received only one injection (7.75%, *p* = 0.0185). In subjects who received a second injection of LBSA0103, the incidence rate of AEs observed up to 12 weeks after the second injection was lower than that observed up to 12 weeks after the first injection (Table 5). Other than the injection site pain, all AEs observed up to 12 weeks after the second injection were considered “unlikely” related to the study drug. There was no noteworthy deviation in the safety trend or the occurrence of AEs when compared to the safety profile of subjects who received the first injection.

**Table 5:** Summary of adverse events before and after the second injection.

	N = 107							
	Before 2^nd^ injection				After 2^nd^ injection			
	Number of subjects (%)		95% CI^†^ [lower, upper]	No. of events	Number of subjects (%)		95% CI^†^ [lower, upper]	No. of events
AE–injection site reaction	8	(7.48)	[3.28, 14.20]	[10]	1	(0.93)	[0.02, 5.10]	[1]
AE–other than injection site reaction	5	(4.67)	[1.53, 10.57]	[5]	1	(0.93)	[0.02, 5.10]	[2]
ADR	7	(6.54)	[2.67, 13.02]	[9]	0	(0.00)	[0.00, 3.39]	[0]
SAE	0	(0.00)	[0.00, 3.39]	[0]	1	(0.93)	[0.02, 5.10]	[1]
Serious ADR	0	(0.00)	[0.00, 3.39]	[0]	0	(0.00)	[0.00, 3.39]	[0]
Unexpected AE	1	(0.93)	[0.02, 5.10]	[1]	1	(0.93)	[0.02, 5.10]	[2]
Unexpected ADR	0	(0.00)	[0.00, 3.39]	[0]	0	(0.00)	[0.00, 3.39]	[0]

AE, adverse event; ADR, adverse drug reaction; SAE, serious adverse event

^†^ 95% CI calculated using the Clopper-Pearson method

To assess the baseline factors associated with the incidence rates of AE, a multiple logistic regression test was performed, and the results showed that the presence of an allergy (OR, 2.00; 95% CI, [1.10, 3.65]; *p* = 0.0234), concurrent medical condition (OR, 1.76; 95% CI, [1.27, 2.44]; *p* = 0.0007), and preexisting/concomitant medication (OR, 3.01; 95% CI, [1.85, 4.89]; *p* < 0.0001), and prior/concomitant therapy (OR, 2.34; 95% CI, [1.49, 3.67]; *p* = 0.0002) were baseline factors that affected the incidence rate of AEs. In addition, the subjects who received two injections of LBSA0103 had a significantly higher incidence rate of AEs than those who received only one injection (OR, 1.86; 95% CI, [1.03, 3.37]; *p* = 0.0398) (Table 6).

**Table 6:** Factors associated with incidence rate of adverse events: Multiple logistic regression analysis.

	Odds ratio	[95% CI]	*p*-value^‡^
Age (continuous)	1.02	[0.99, 1.04]	0.1408
Presence of allergy (1 = yes, 0 = no)	2.00	[1.10, 3.65]	0.0234
Preexisting medical condition (1 = yes, 0 = no)	1.41	[0.95, 2.10]	0.0874
Concurrent medical condition (1 = yes, 0 = no)	1.76	[1.27, 2.44]	0.0007
Preexisting/concomitant medication (1 = yes, 0 = no)	3.01	[1.85, 4.89]	<0.0001
Prior/concomitant therapy (1 = yes, 0 = no)	2.34	[1.49, 3.67]	0.0002
Aged ≥65 years (1 = yes, 0 = no)	1.02	[0.65, 1.62]	0.9225
Second injection (1 = yes, 0 = no)	1.86	[1.03, 3.37]	0.0398

^‡^ Calculated using multiple logistic regression analysis

**Fig 3:**
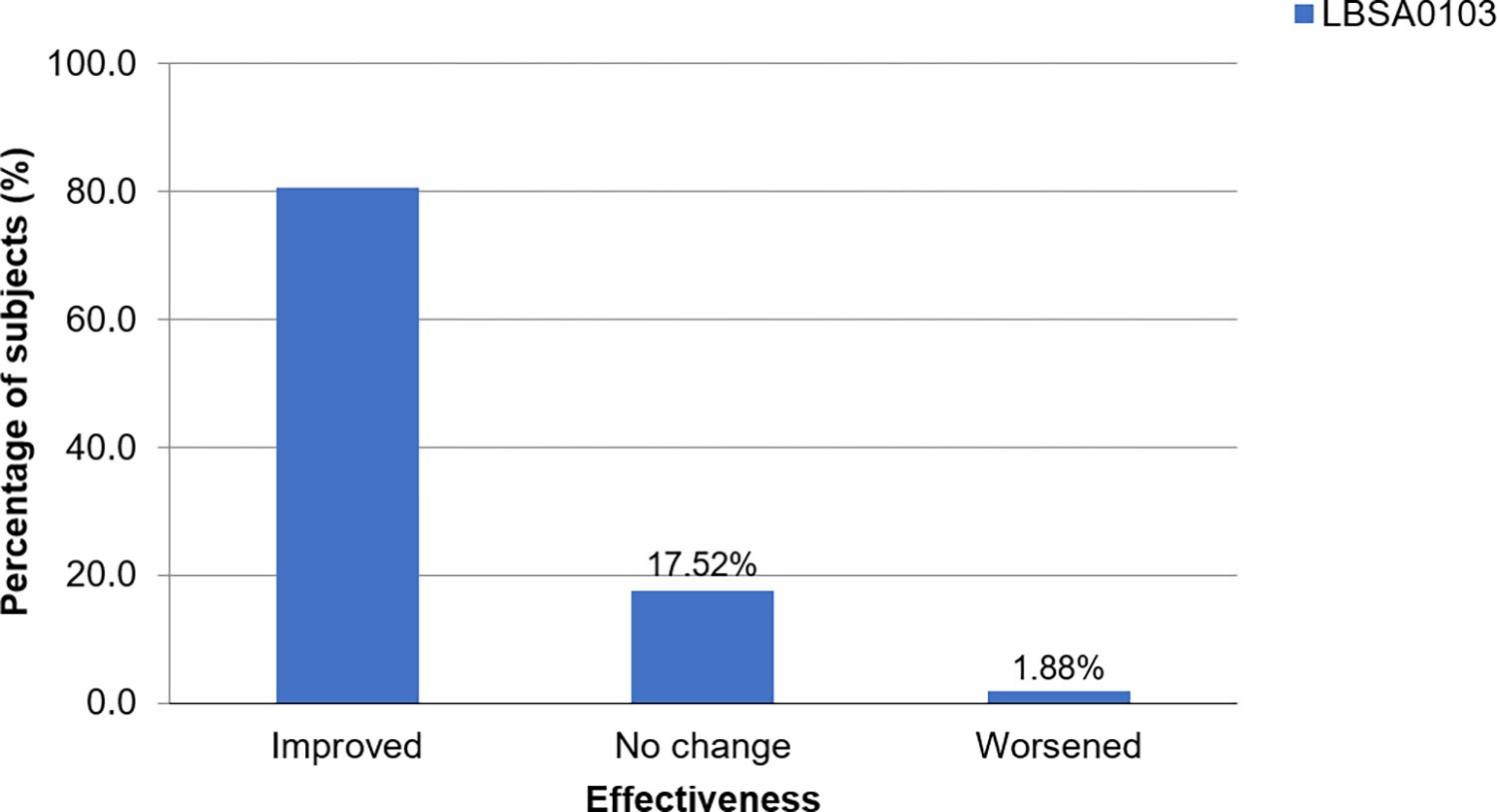
Effectiveness assessment at 12 weeks after the last injection (effectiveness analysis set). Based on an improvement scale ranging from “improved” to “undeterminable”.

*3*.*2*.*2*.*1 Effectiveness analysis*. Among 3,003 subjects, effectiveness data, as assessed by the investigators, were collected from 2,986 subjects. Of these, the condition of 80.61% of the subjects was assessed as “improved” at 12 weeks after the last injection, demonstrating the therapeutic effectiveness of LBSA0103 (Fig 3). In subgroup analyses, the effectiveness of LBSA0103 was lower in subjects with concurrent medical conditions than those without (74.41% vs. 84.93%, *p* < 0.0001), subjects who used preexisting/concomitant medication than those who did not (77.39% vs. 86.84, *p* < 0.0001), and subjects with hepatic impairment (68.09% vs. 80.81%, *p* = 0.0286) than those without. Other baseline factors showed no significant effects on the effectiveness of the study drug.

**Fig 4:**
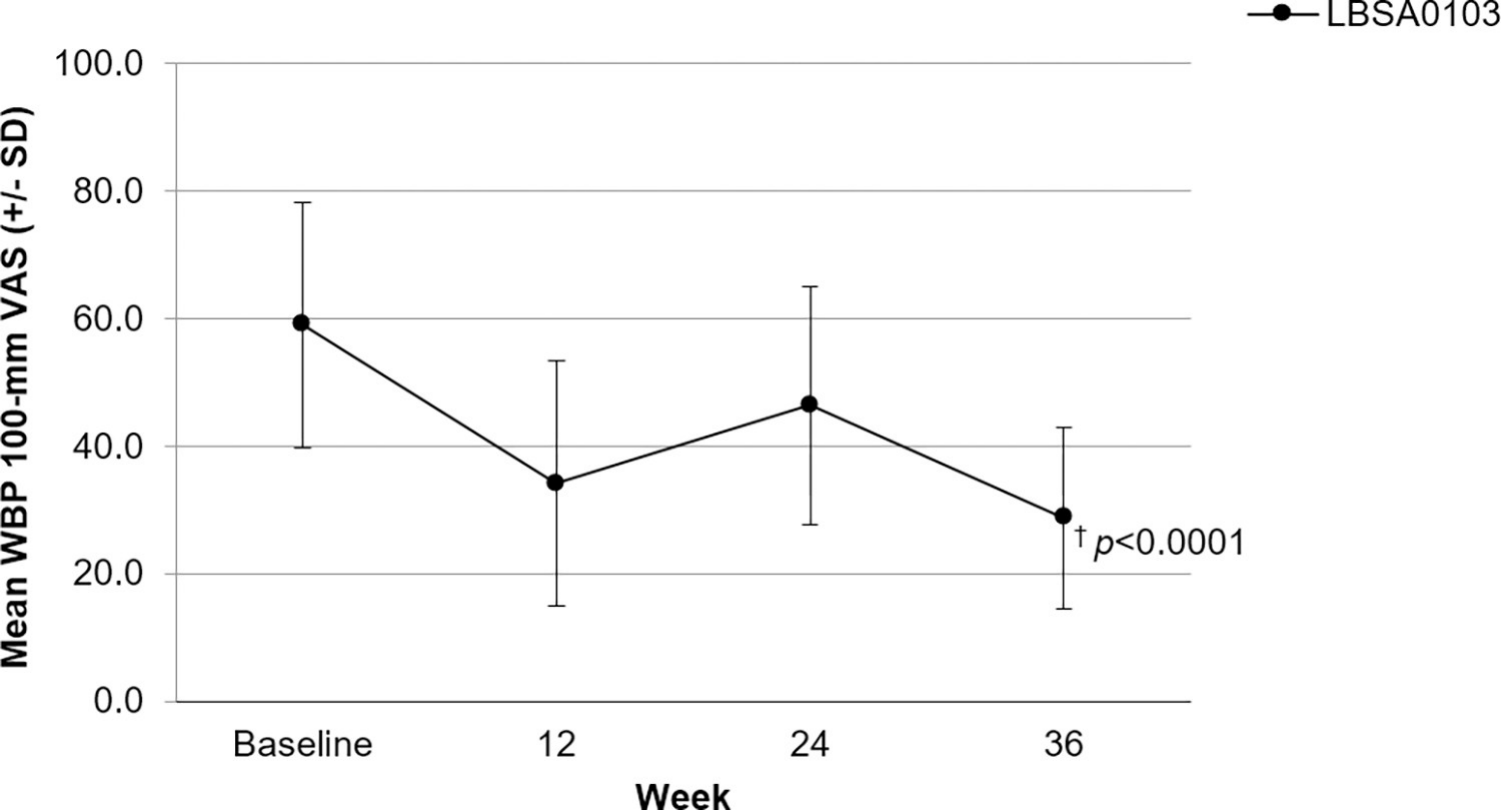
WBP using a 100-mm VAS over time. WBP, weight-bearing pain; VAS, Visual analog scale; SD, standard deviation. Data are expressed as the mean ± standard deviation (SD). * A total of 107 subjects received a second injection of LBSA0103 at least 24 weeks apart from the first injection. ^†^ p<0.0001.

The improvement in WBP, based on the 100-mm VAS score, over the PMS period is shown in Fig 4. LBSA0103 showed a clinically significant (*p* < 0.0001) reduction in WBP at 12 weeks after the first and second injections. Subjects had a mean (± SD) VAS score of 58.98 (± 19.29) mm at baseline. The mean (SD) reduction in WBP from baseline to 12 weeks after the first injection was 24.79 (± 20.55) mm. Subjects assessed just before the second injection (24 weeks apart from the first injection) had a mean (± SD) VAS score of 46.38 (± 18.65) mm. At 12 weeks after the second injection, the mean (± SD) reduction in VAS score was 17.63 (±12.31) mm. Changes in VAS scores were compared in subjects grouped by unilateral or bilateral involvement of the knee. A clinically significant reduction in VAS scores was observed in both the subject groups (*p* < 0.0001).

To assess the baseline factors associated with the effectiveness of LBSA0103, multiple logistic regression analysis was performed, and the results showed that concurrent medical conditions (OR, 0.62; 95% CI, [0.50, 0.76;] *p* < 0.0001) and preexisting/concomitant medication (OR, 0.70; 95% CI, [0.54, 0.89]; *p* = 0.004) were associated with increased effectiveness of the study drug. Furthermore, the effectiveness was significantly higher in subjects treated for bilateral knee OA than in those treated for unilateral knee OA (OR, 0.79; 95% CI, [0.66, 0.96], *p* = 0.0175) (Table 7).

**Table 7:** Factors associated with effectiveness: Multiple logistic regression analysis.

	Odds ratio	[95% CI]	*p*-value^‡^
Concurrent medical condition (1 = yes, 0 = no)	0.62	[0.50, 0.76]	<0.0001
Preexisting/concomitant medication (1 = yes, 0 = no)	0.70	[0.54, 0.89]	0.0040
Presence of hepatic impairment (1 = yes, 0 = no)	0.71	[0.38, 1.33]	0.2856
Number of knee OA (1 = unilateral, 0 = bilateral)	0.79	[0.66, 0.96]	0.0175

^‡^ Calculated using multiple logistic regression analysis

## 4. Discussion

The safety and effectiveness of LBSA0103 have been well established by controlled randomized trials conducted with LBSA0103 [21, 22]. This 6-year PMS study provides robust evidence to refine the safety and effectiveness of LBSA0103 in a real-world setting. More than 3,000 subjects were monitored for at least 12 weeks after one or two IA injections in routine clinical practice. Overall, AEs and ADRs occurred in 7.96% and 3.18% of subjects, respectively. Injection site pain was the most common ADR and no serious ADRs were reported during the entire study period. LBSA0103 was also shown to be effective when assessed based on an improvement index from “improved” to “undeterminable” and in terms of reduction in WBP using a 100-mm VAS. Furthermore, the findings of our study were mostly consistent with the observations made during clinical development as well as with findings of other studies conducted in various populations with HMW-HA [21, 22, 25–28]. There were no new findings that would raise questions about the safety of LBSA0103 in real-world usage, and its effectiveness was shown to be comparable with the results obtained from clinical studies at the time of approval. The significance of this study is that it included a large number of subjects to detect very rare or uncommon AEs that might not have been reported in previous clinical trials of LBSA0103. To the best of our knowledge, this is the first PMS study to assess the safety and effectiveness of HMW-HA in a large patient population. PMS studies have previously been conducted for HMW-HAs; however, they did not involve a sufficient number of subjects to identify previously unrecognized AEs [29, 30].

The reported incidence of AEs following administration of HMW-HA varies considerably across studies; these differences can be attributed to factors such as different study designs, baseline characteristics, and methods of defining and collecting AEs. However, the types of AEs reported in our study are consistent with those reported in the previous clinical studies conducted with LBSA0103 [21, 22, 31]. Studies have shown that injection site pain was consistently the most common AE, which is in line with the observations made in our study [21, 22, 31]. In studies conducted on other HMW-HAs, arthralgia, joint swelling, joint effusion, and joint stiffness have been reported [25–28]. These AEs were also reported in our study but were not as frequently reported as that in studies conducted on HMW-HA [25–28].

There also have been reports about the development of pseudoseptic reactions in patients receiving HMW-HAs [10]. Yoshioka et al. speculated that the pseudoseptic reactions reported in patients receiving Synvisc^®^, a cross-linked HA derived from rooster comb, are caused due to its unique cross-linking structure via the protein and DVS and/or (1→3)-β-D-glucan content present in the formulation [11]. Similarly, Ishikawa et al. has suggested that the differences in biocompatibility and immunogenicity between two cross-linked HA products are due to differences in cross-linking technology [16]. No pseudoseptic reactions were reported during the entire PMS period. Based on the literature findings above, we can propose that the safety profile of LBSA0103 observed in this study can partly be attributed to the cross-linking agent, BDDE, used in LBSA0103 formulation. BDDE cross-linked HAs have been shown to be safe and have not demonstrated any cytotoxicity [20].

In the subgroup analyses, subjects aged ≥65 years were associated with a higher incidence rate of AEs. The effects of LBSA0103 in geriatric patients did not reveal any significant differences compared with that noted in younger subjects [21, 22]. The increased AE incidence rate in our study might be due to age-related physiological changes, which result in altered drug pharmacokinetics and pharmacodynamics [32]. Polypharmacy, multimorbidity, and other age-related factors must be considered when interpreting safety data obtained in this population. However, given that the incidence rates of ADRs were similar in subjects ≥65 years when compared with the incidence rates in the overall study population, with injection site pain being the most frequently reported ADR in both the groups, we can expect the safety profile of LBSA0103 to be comparable. Most importantly, age was not a significant factor associated with the incidence rate of AE when tested in a multiple logistic regression analysis. Therefore, LBSA0103 can be safely used in elderly patients aged ≥65 years. In fact, Maheu et al. claimed that IA-HA is a good treatment option for older patients receiving polypharmacy due to the low potential for AEs and the drug-drug interactions with IA-HA [33, 34]. Further, in the multiple logistic regression analysis, subjects with an allergy, concurrent medical conditions, and preexisting/concomitant medication and prior/concomitant therapy were associated with a significantly higher incidence rate of AEs. However, it is difficult to draw any clinically meaningful significant conclusions from these findings. Medications taken and therapies received prior to and during the study period might have affected the incidence rate of AEs and, hence, the safety outcomes of this study. Medications administered to treat AEs may also have an impact on safety outcomes. Well-controlled studies are needed to further corroborate these findings. In addition, the subjects who received two injections of LBSA0103 were associated with a significantly higher incidence rate of AEs when compared with those who received only one injection. This is expected because increased drug exposure is often associated with an increased risk of AEs. However, only a small fraction (107 subjects, 3.41%) of subjects received the second injection; hence, this is not sufficient to support any claims. Moreover, repeated IA injection (26 weeks apart) did not increase the risk of ADRs according to a previous study conducted with LBSA0103 [22]. In studies conducted on other HMW-HAs, no increase was reported in the risk of AE in patients receiving a second injection [26, 27, 35] Therefore, the findings in our study further support the safety of added injections of LBSA0103 for knee OA.

In terms of effectiveness, the results of randomized clinical trials conducted with HMW-HAs have not been uniformly positive, and whether the MW of an HA influences efficacy is still controversial [25–28, 36–42]. There is also discordance among multiple treatment guidelines regarding the use of IA-HA [25–28, 36–42]. Maheu et al. suggested that this discordance is due to the modest effect size for IA-HA found in randomized controlled trials [33]. However, the compiled available evidence suggests that IA-HA is effective and beneficial in terms of pain relief in knee OA [21, 26, 39–42]. A recent meta-analysis showed that IA-HA products with an average MW of ≥300 kDa provide superior efficacy and safety [41]. Strand et al. concluded that an IA injection of US-approved viscosupplements for 26 weeks is safe and efficacious in patients with symptomatic knee OA [43]. A retrospective observational study has reported that patients with knee OA who were treated with IA-HA had delayed progression to total knee replacement when compared with those who did not receive the injection [7]. In line with the findings of these studies, LBSA0103 was shown to be effective in reducing pain at 12 weeks after the last injection in our study. The magnitude of pain reduction observed in our study also does not seem to differ significantly from that reported in earlier studies of LBSA0103 and in studies conducted with other preparations of HMW-HA [21, 22, 35, 44]. Additionally, factors associated with the effectiveness of the study drug were also analyzed. As a result, a tendency toward increased effectiveness following administration of LBSA0103 was shown in subjects without a concurrent medical condition and in those who did not take any preexisting/concomitant medication. Concurrent illness or medical condition can alter the baseline status of the subjects and, hence, the effectiveness of the drug. It is also noteworthy that the study drug showed better effectiveness in subjects who did not take any preexisting/concomitant medications. Subjects who were taking anti-inflammatory and antirheumatic drug products accounted for more than 50% of the total subjects, which was suggestive of the fact that their OA condition and level of pain were more severe, resulting in reduced effectiveness of the study drug. However, the results should be interpreted with caution, as it is an uncontrolled real-world study. Bias cannot be completely excluded in this type of study, and other confounding factors, unknown or not considered in our statistical analysis, may have affected the outcome of our study. Furthermore, subjects who were treated for bilateral knee OA were associated with significantly higher effectiveness when compared with treated for unilateral knee OA. This is because improvements in both the knees are likely to lead to a more satisfactory response from subjects when compared with that in subjects who felt improvement only in one knee.

There were limitations to our study. First, it was an open-label, single-arm, non-comparative, and non-confirmatory study and as we had no control group for comparison, the safety and effectiveness of LBSA0103 could not be compared with those who received no or other treatments. Nonetheless, the study included a large number of subjects to identify any previously unrecognized AEs and the data obtained provided sufficient evidence for the safety of LBSA0103. Moreover, this study design had the advantage of being closer to the real-world clinical setting. Second, the effectiveness endpoints in our study only included parameters associated with pain. Other experimental measures or scores to quantify the effectiveness of LBSA0103 would have further characterized the efficacy of using LBSA0103 for knee OA. Third, the study included only Asian patients, predominantly female; therefore, a possible racial or sex disparity may be present. Further studies, which include other racial and ethnic groups, are required before making any generalization about the results of our study. Fourth, as this was not a controlled trial, other factors such as concomitant medications and therapies may have introduced bias and may have affected the outcomes of our study. Factors such as incomplete reporting information, under-reporting, biased reporting, and difficulties in attributing an AE to the investigational drug are also limitations of uncontrolled, post-marketing surveillance study. The present results support the safety and effectiveness of LBSA0103, but they must be interpreted with care. Fifth, in terms of safety assessment, only treating physicians, all of them who were orthopedics, were involved. Additional input from general practitioners would have been beneficial in improving the quality of safety data generated in this study. Lastly, the follow-up period of LBSA0103 (24 weeks) in this study was relatively short. Additional randomized controlled trials with longer follow-ups may be helpful to investigate the long-term safety of LBSA0103, especially any adverse events associated with delayed-onset.

Based on the findings of our study, we conclude that LBSA0103 has an acceptable benefit/risk profile when administered to patients aged ≥19 years for symptomatic relief of knee OA. Potential AEs noted after administration of LBSA0103 will be continued to be monitored through routine safety reporting channels.

## Supporting information

S1 Appendix List of IRBs. (XLSX)

S1 Table Adverse events and adverse drug reaction in overall population. (XLSX)

S2 Table Serious adverse events and serious adverse drug reaction in overall population. (XLSX)

S3 Table Unexpected adverse events and unexpected adverse drug reactions in overall population. (XLSX)

S4 Table Outcomes of adverse events in overall population. (XLSX)

S5 Table Causality of adverse events in overall population. (XLSX)

S6 Table Summary of adverse events in geriatrics. (XLSX)

S7 Table Adverse events and adverse drug reaction in geriatrics. (XLSX)

S8 Table Summary of adverse events in special populations. (XLSX)

S9 Table Adverse events depending on injection site and after the 2nd injection. (XLSX)

S10 Table 100mm VAS change before and after the injection. (XLSX)

S11 Table Effectiveness outcome after the final injection. (XLSX)
